# Dynamic MicroRNA Expression Profiles During Embryonic Development Provide Novel Insights Into Cardiac *Sinus Venosus/*Inflow Tract Differentiation

**DOI:** 10.3389/fcell.2021.767954

**Published:** 2022-01-11

**Authors:** Carlos Garcia-Padilla, Angel Dueñas, Diego Franco, Virginio Garcia-Lopez, Amelia Aranega, Virginio Garcia-Martinez, Carmen Lopez-Sanchez

**Affiliations:** ^1^ Department of Human Anatomy and Embryology, Faculty of Medicine, Institute of Molecular Pathology Biomarkers, University of Extremadura, Badajoz, Spain; ^2^ Department of Experimental Biology, University of Jaen, Jaen, Spain; ^3^ Fundación Medina, Granada, Spain

**Keywords:** heart development, microRNAs, expression profiles, Hox genes, sinus venosus, inflow tract

## Abstract

MicroRNAs have been explored in different organisms and are involved as molecular switches modulating cellular specification and differentiation during the embryonic development, including the cardiovascular system. In this study, we analyze the expression profiles of different microRNAs during early cardiac development. By using whole mount *in situ* hybridization in developing chick embryos, with microRNA-specific LNA probes, we carried out a detailed study of miR-23b, miR-130a, miR-106a, and miR-100 expression during early stages of embryogenesis (HH3 to HH17). We also correlated those findings with putative microRNA target genes by means of mirWalk and TargetScan analyses. Our results demonstrate a dynamic expression pattern in cardiac precursor cells from the primitive streak to the cardiac looping stages for miR-23b, miR-130a, and miR-106a. Additionally, miR-100 is later detectable during cardiac looping stages (HH15-17). Interestingly, the *sinus venosus*/inflow tract was shown to be the most representative cardiac area for the convergent expression of the four microRNAs. Through *in silico* analysis we revealed that distinct Hox family members are predicted to be targeted by the above microRNAs. We also identified expression of several Hox genes in the *sinus venosus* at stages HH11 and HH15. In addition, by means of gain-of-function experiments both in cardiomyoblasts and *sinus venosus* explants, we demonstrated the modulation of the different Hox clusters, Hoxa, Hoxb, Hoxc, and Hoxd genes, by these microRNAs. Furthermore, we correlated the negative modulation of several Hox genes, such as Hoxa3, Hoxa4, Hoxa5, Hoxc6, or Hoxd4. Finally, we demonstrated through a dual luciferase assay that Hoxa1 is targeted by miR-130a and Hoxa4 is targeted by both miR-23b and miR-106a, supporting a possible role of these microRNAs in Hox gene modulation during differentiation and compartmentalization of the posterior structures of the developing venous pole of the heart.

## Introduction

During chick gastrulation (stage HH3; Hamburger and Hamilton, 1951, 1992) the primitive streak precardiac cells invaginate and migrate anterolaterally to form the precardiac mesoderm, between the ectoderm and the inductive adjacent endoderm (Garcia-Martinez and Schoenwolf, 1993; Schultheiss et al., 1995; Garcia-Martinez et al., 1997; Redkar et al., 2001; Lopez-Sanchez et al., 2001, 2009, 2018), at both sides of the embryo (stages HH5-7), determining the first heart field (FHF; Harvey, 2002). Subsequently, this field will form the primitive endocardial tubes (stage HH8), which in the midline fuse into a single heart tube (stage HH10), structured into the endocardial and myocardial layers (Lopez-Sanchez and Garcia-Martinez 2011). Later on, a progenitor population originating from the adjacent pharyngeal mesoderm, the secondary heart field (SHF; Buckingham et al., 2005), will contribute to further cardiac development, giving rise to the dorsal *mesocardium*, as well as the posterior (venous) and anterior (arterial) heart poles, differentiating the inflow (IFT) and outflow tracts (OFT), respectively (Waldo et al., 2005; Abu-Issa and Kirby 2008; van den Berg et al., 2009; Camp et al., 2012; Zaffran and Kelly, 2012; De Bono et al., 2018a). The IFT, a relevant segment in the sinoatrial node formation, develops from a restricted set of cardiomyocytes located in the *sinus venosus* (SV). Furthermore, the outer surface of the SV gives rise to the proepicardium—epicardial primordium—development (Christoffels et al., 2006; van Wijk et al., 2009, 2018; Mommersteeg et al., 2010; Carmona et al., 2018).

A complex network of signaling pathways and transcriptional factors is required to regulate early cardiac development. In this sense, the FGF, TGF, and Wnt pathways, among others, have been widely involved in early cardiogenesis. In particular, within this signaling network, retinoic acid (RA) has been shown to pattern the SHF (Hochgreb et al., 2003; Stefanovic and Zaffran, 2017). RA signaling is required for SHF differentiation, modulating the expression of specific marker genes, including Fgf8, Fgf10, and Tbx1 (Ryckebusch et al., 2008; Sirbu et al., 2008; De Bono et al., 2018b). Previous genetic lineage analyses in mice have revealed that Homeobox (Hox) gene expressions—Hoxa1, Hoxa3, and Hoxb1—define distinct domains and sub-domains within the SHF (Bertrand et al., 2011). Thus, Hoxa1- and Hoxb1-expressing progenitor cells contribute to both cardiac poles’ development, the IFT and the inferior wall of the OFT (Bertrand et al., 2011; Lescroart and Zaffran, 2018; Stefanovic et al., 2020).

Due to the fact that microRNAs represent a novel layer of complexity in the regulatory networks controlling gene expression, cell specification, and differentiation (Choi et al., 2013; Rajabi et al., 2020), this subclass of non-coding RNAs has been widely analyzed by its relevant role in cardiac development. Previous studies have provided evidence on the differential expression of several microRNAs contributing to cardiac fate specification and maintenance of cardiac progenitors during development (Chinchilla et al., 2011; Cao et al., 2012; Lopez-Sanchez et al., 2015a; Lopez-Sanchez et al., 2015b; Yan and Jiao 2016; Kalayinia et al., 2021). Some microRNA expressions, such as those of miR-125b, miR-142-3p, and miR-137, have also been referenced in the SV/IFT (Darnell et al., 2006).

In order to shed light on microRNAs and their role during early cardiac development, in this work, we analyze the expression pattern of miR-23b, miR-130a, miR-106a, and miR-100, from early stages of embryogenesis (HH3 to HH17). Although these microRNAs have been previously involved in cardiac structural and functional characteristics (Sucharov et al., 2008; Thum et al., 2008; Aguirre et al., 2014; Guan et al., 2016; Boureima Oumarou et al., 2019), their roles during cardiac development have still not been assessed. Our results show a common expression of these microRNAs in specific cardiac structures, the SV/IFT being the most representative cardiac area for their convergent expression. Also, we have correlated these findings with several Hox family members revealed as targets of these microRNAs by means of mirWalk and TargetScan analyses. Furthermore, we have identified that several Hox genes targeted by these microRNAs are expressed in the HH11 and HH15 *sinus venosus*. Finally, by using *in vitro* microRNA gain-of-function experiments on cardiomyoblasts derived from undifferentiated H9c2 cells, together with an analysis of chicken *sinus venosus* explants, we have shown in this study the specific relevance of distinct microRNAs in Hox gene modulation, suggesting a possible molecular role in the differentiation and/or compartmentalization of the developing venous pole of the heart.

## Results

### Expression Profile of miR-23b, miR-130a, miR-106a, and miR-100 During Early Embryonic Development

**FIGURE 1:**
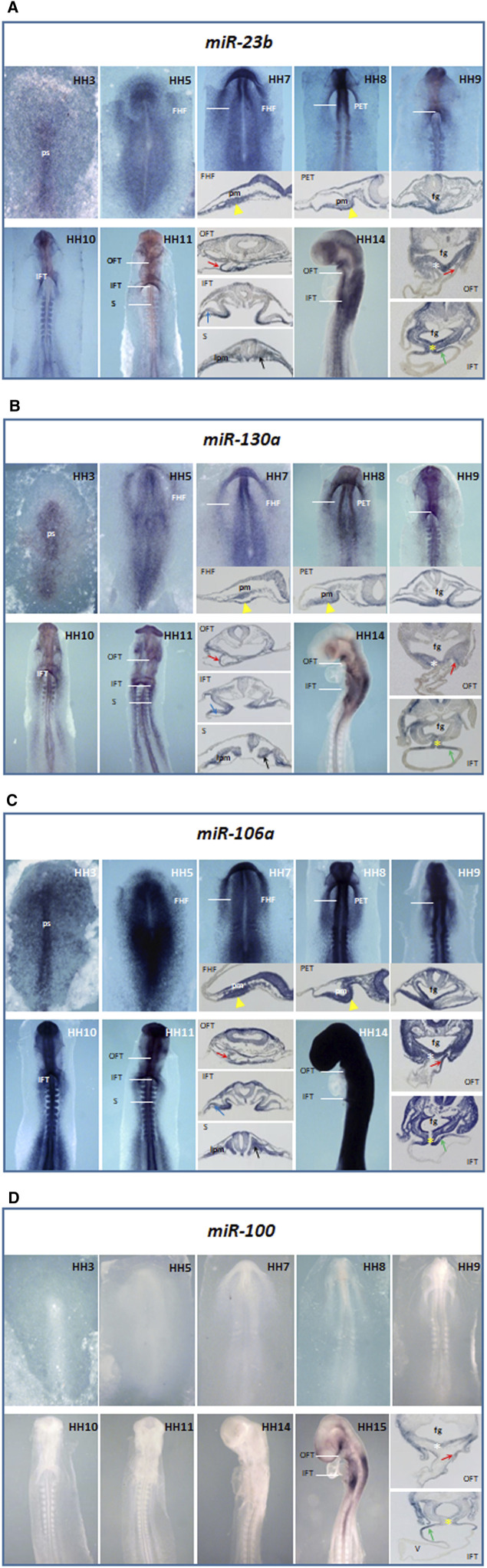
Whole-mount ISH analysis of miR-23b **(A)**, miR-130a **(B)**, miR-106a **(C)**, and miR-100 **(D)** during chick embryo cardiac development, from HH3 through HH14. White lines indicate the transverse section levels of selected embryos: FHF, first heart field; PET, primitive endocardial tube; IFT, inflow tract; OFT, outflow tract and S, somite level. Note their expressions in the primitive streak (ps), precardiac mesoderm (pm), adjacent endoderm to precardiac mesoderm (yellow arrowhead), OFT (red arrow), dorsal surface of *sinus venosus* (green arrow), dorsal mesocardium (yellow asterisk), and vitelline vein (blue arrow); as well as in the foregut (fg), pharyngeal mesoderm (white asterisk), lateral plate mesoderm (lpm), and somites (black arrow) for miR-23b **(A)**, miR-130a **(B)**, and miR-106a **(C)**. Just from stage HH15 is observable miR-100 expression **(D)** in cardiac structures. V, ventricle.

**FIGURE 2:**
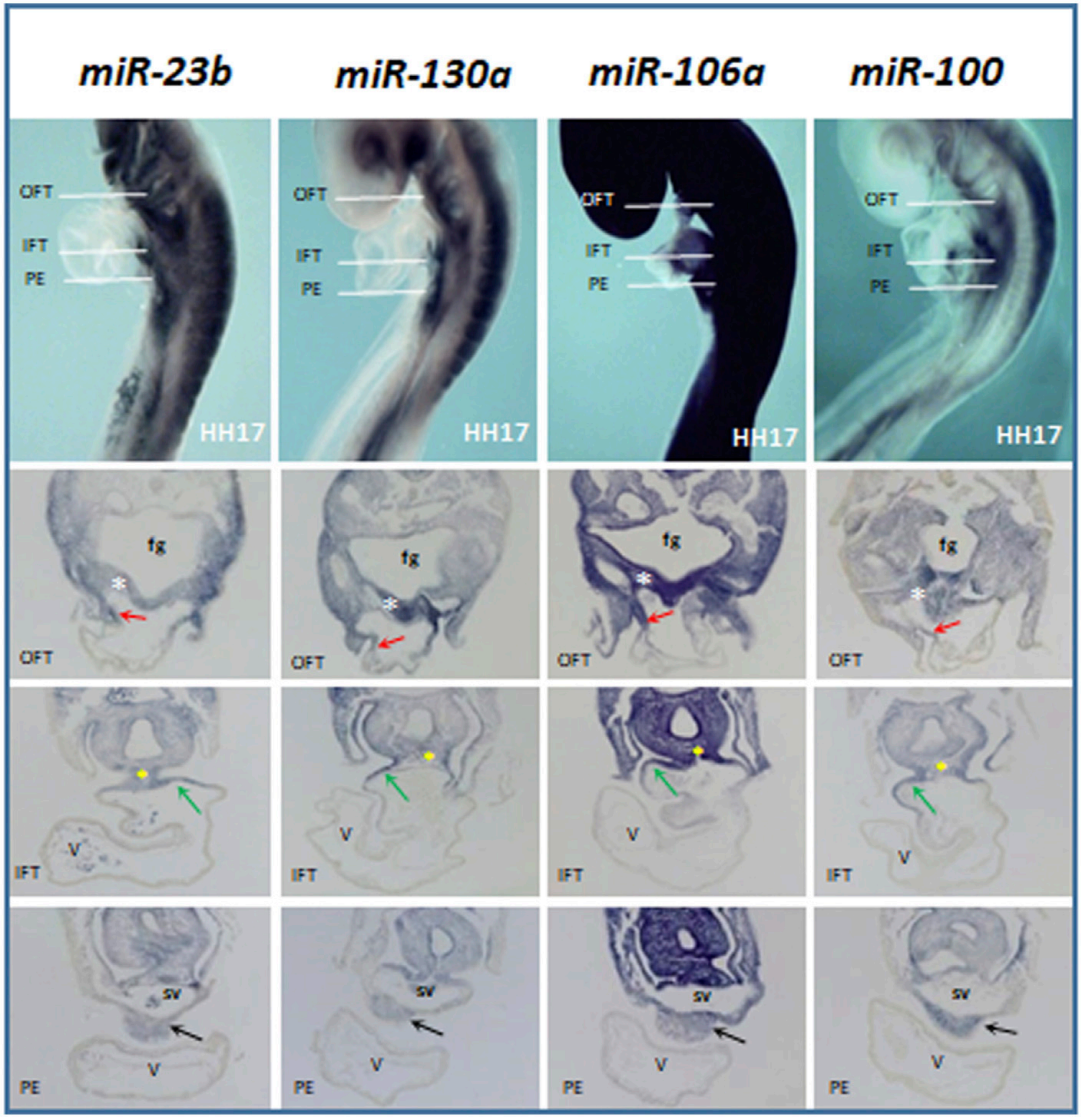
Comparative whole-mount ISH analysis of miR-23b, miR-130a, miR-106a, and miR-100 expression profiles at stage HH17. The transverse section levels are indicated with white lines: OFT, outflow tract; IFT, inflow tract, and PE, proepicardium. Note their expressions in the lateral–dorsal wall of the OFT (red arrow), foregut (fg), dorsal wall of the IFT (green arrow), dorsal mesocardium (yellow asterisk), and proepicardium (black arrow). White asterisk, pharyngeal mesoderm; V, ventricle; sv, *sinus venosus*.

In this work, we have analyzed the expression profile of miR-23b, miR-130a, miR-106a, and miR-100 during early chicken embryonic development (Figures 1, 2), starting at early gastrulation stages through the formation of the early cardiac looped stages, from HH3 to HH17.

From early stages of development, miR-23b (Figure 1A) extends along the primitive streak (HH3). Later, this expression expands to the precardiac mesoderm and the underlying endoderm at both sides of the embryo and contributes to the formation of the first heart field (HH5), maintaining its expression in both primitive endocardial tubes (HH8). As shown in HH9-10, during cardiac tube formation, this expression is progressively restricted to the inflow tract. Noticeably, during early cardiac looping (HH11), miR-23b is expressed in both inflow and outflow tracts, subsequently being more evident on the dorsal surface of the cardiac *sinus venosus* and the dorsal mesocardium (HH14).

We have previously described that miR-130a starts expressing at the primitive streak stage (HH3), followed by an expansion toward both sides of the mesodermal and endodermal layers of the embryo (HH4). From stage HH5 onward, miR-130a is progressively restricted to the precardiac mesoderm and its underlying endoderm, subsequently expressing in both primitive endocardial tubes (Lopez-Sanchez et al., 2015a). One of the novelties of this study lies in the identification of miR-130a expression during primitive cardiac tube formation (Figure 1B), specifically in the dorsal mesocardium and the dorsal wall of the cardiac *sinus venosus* (HH9-14), as well as in the outflow tract.

During early stages of development, miR-106a (Figure 1C) shows a broad expression in the embryo, sharper in the primitive streak (HH3). Afterward, it expresses homogeneously throughout the embryo (HH5-13), excluding the extraembryonic tissue. Although this expression covers all the heart regions and derivatives at early stages (HH8-11), it is restricted to the dorsal mesocardium and the inflow and outflow tracts at later stages (HH14). The signal stays homogeneously strong in the remaining embryo, except in the developing cardiac chambers.

Additionally, we have observed the expression of the microRNAs mentioned above in somites and the lateral plate mesoderm, among others (Figures 1A–C).

Unlike miR-23b, miR-130a, and miR-106a, the expression of miR-100 starts at the HH15 stage, being evident on the dorsal surface of the inflow tract and in the dorsal mesocardium and outflow tract (Figure 1D).

During cardiac looping stages (HH15–17), the four microRNAs analyzed (Figure 2) are expressed in the lateral–dorsal wall of the outflow tract and foregut, being evident in the dorsal wall of the inflow tract and the dorsal mesocardium. Interestingly, in these stages, all these microRNAs show a marked expression in the proepicardium, which is formed at the cardiac inflow region, both coming from a common progenitor pool (van Wijk et al., 2009, 2018).

### Hox Cluster Family Members Are Differentially Modulated by miR-23b, miR-130a, miR-106a, and miR-100

**FIGURE 3:**
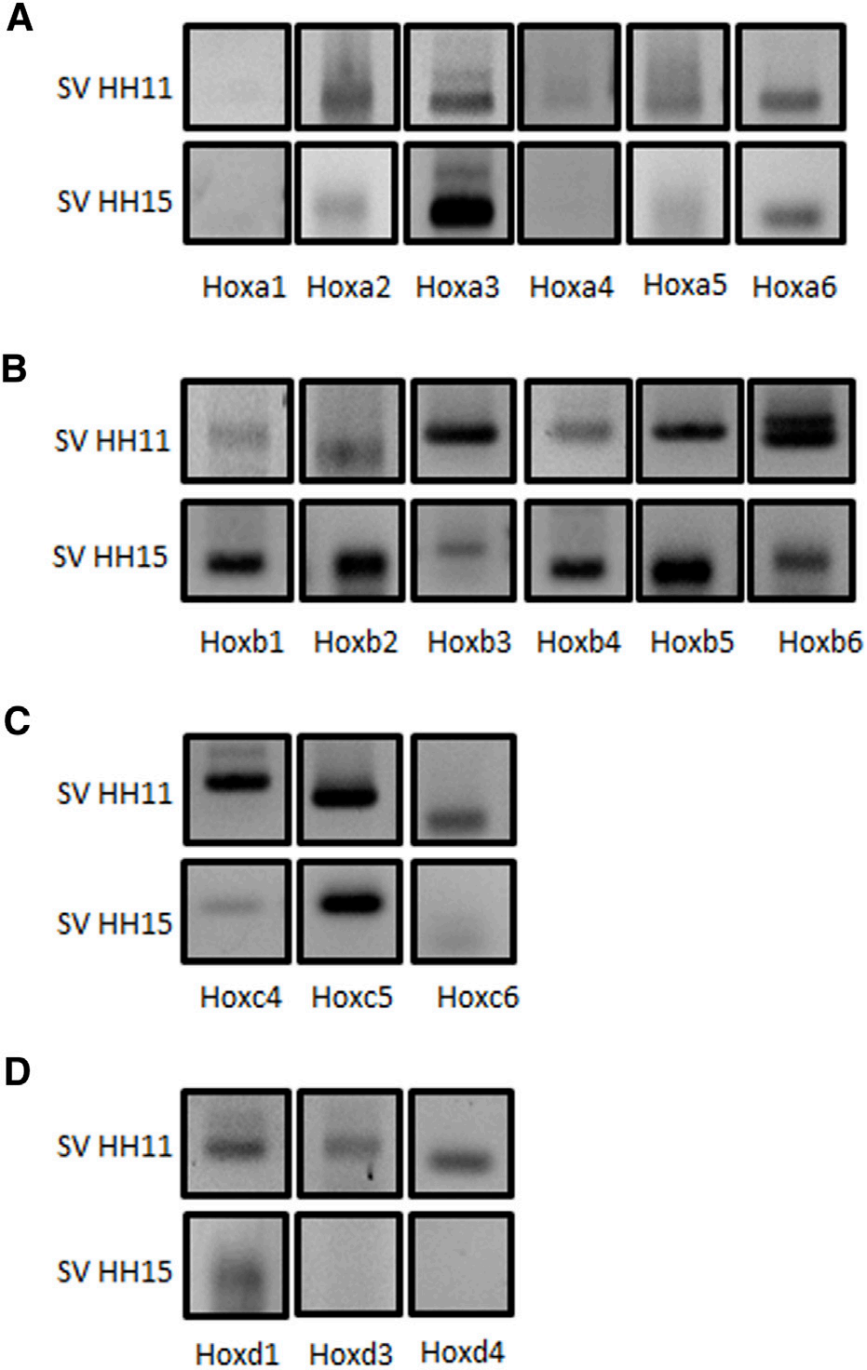
Comparative analysis of Hox gene expression in the *sinus venosus* between stages HH11 and HH15. RT-PCR analyses of Hoxa (Panel **A**), Hoxb (Panel **B**), Hoxc (Panel **C**), and Hoxd (Panel **D**) cluster family members, as demonstrated by their amplified amplicons on agarose gel electrophoresis. Observe that most Hox genes analyzed, except Hoxa1, are detectable at HH11 and HH15 in the *sinus venosus*.

Modulation by multiple microRNAs within the same pathway—and even common targets—has been widely reported (Wu et al., 2010; Shu et al., 2017). Since those microRNAs analyzed in this study are particularly expressed in the cardiac inflow tract at early developing stages, we have considered that they could recognize common targets likely involved in the differentiation processes of this segment. Several gene families and transcription factors are important to establish compartmentalization of the embryo, especially during early stages of development. In this sense, Hox gene families have gained pivotal relevance in the last 3 decades. Those genes are broadly conserved during evolution, and their roles to shape the body of the embryo have been widely described in multiple species (Roux and Zaffran, 2016; Lescroart and Zaffran, 2018). In addition, expression of Hox genes in the cardiovascular system is rather restricted, mostly confined to the posterior SHF-derived venous pole of the heart. We therefore explored the expression profile of all cranially expressed Hox genes, including all paralogues (Hoxa to Hoxd) from one to six in the chicken cardiac *sinus venosus* at stages HH11 and HH15 (Figure 3). From our data, most of these Hox genes were detected in the *sinus venosus* at stages HH11 and HH15, except for Hoxa1 (Figure 3A). In addition, we observed that Hoxa2, Hoxa4, and Hoxa5 (Figure 3A), Hoxb3 and Hoxb6 (Figure 3B), Hoxc4 and Hoxc6 (Figure 3C), and Hoxd1, Hoxd3, and Hoxd4 (Figure 3D) display well-marked expression at stage HH11 compared to HH15. On the other hand, Hoxa3 (Figure 3A), Hoxb1, Hoxb2, Hoxb4, and Hoxb5 (Figure 3B) showed an intense expression at HH15. Finally, Hoxa6 and Hoxc5 presented a similar expression at both stages (Figures 3A,C).

**FIGURE 4:**
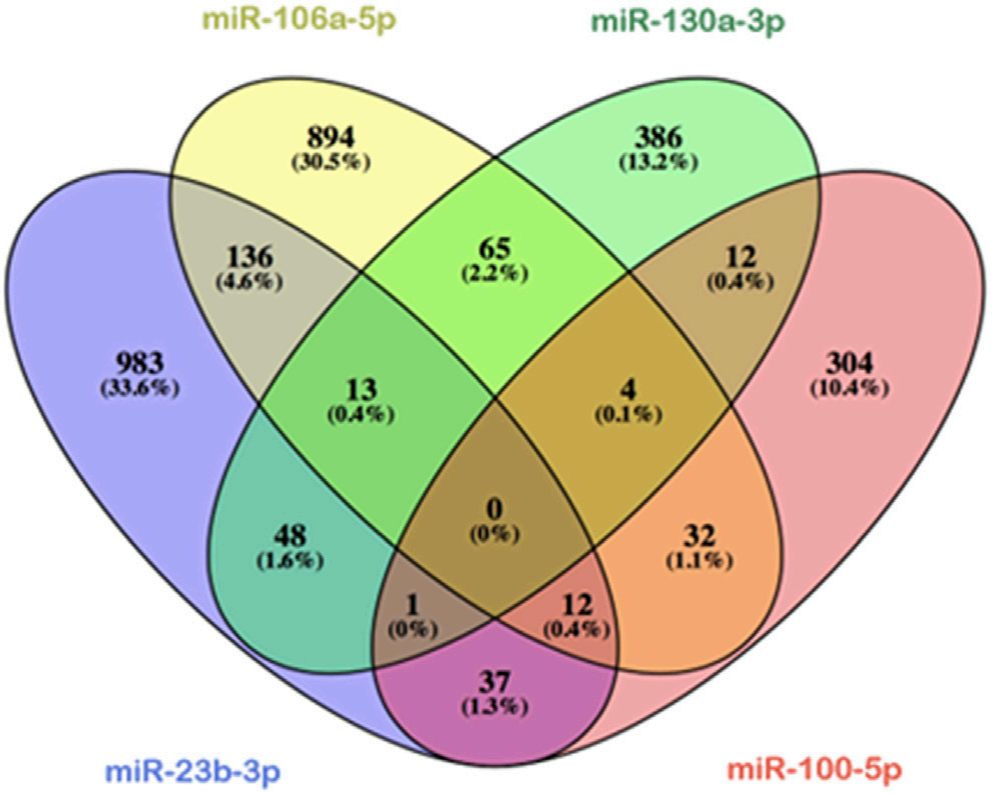
Venn diagram shared potential targets of miR-23b-3p (blue ellipse), miR-130a-3p (green ellipse), miR-106-5p (yellow ellipse), andmiR-100-5p (red ellipse), studied by means of mirWalk software analysis. Note *in silico* predictions that the four microRNAs studied have not shared any target, while a few numbers of common targets are recognized by three of the four microRNAs. Overlapping ellipses represent shared targets between these microRNAs.

Since several Hox genes are expressed at stages HH11 and HH15, we considered in this study the possibility that microRNAs under analysis could be recognizing the 3′UTRs of the distinct Hox genes and thus modulating their expression. By using mirWalk (Sticht et al., 2018) and TargetScan softwares, we analyzed *in silico* those common targets shared by miR-23b, miR-130a, miR-106a, and miR-100. It is observed that these four microRNAs do not share any targets simultaneously, as shown in Figure 4. On the other hand, all Hox genes are recognized by at least one of these microRNAs, except Hoxa6 (Supplementary Table S1).

**FIGURE 5:**
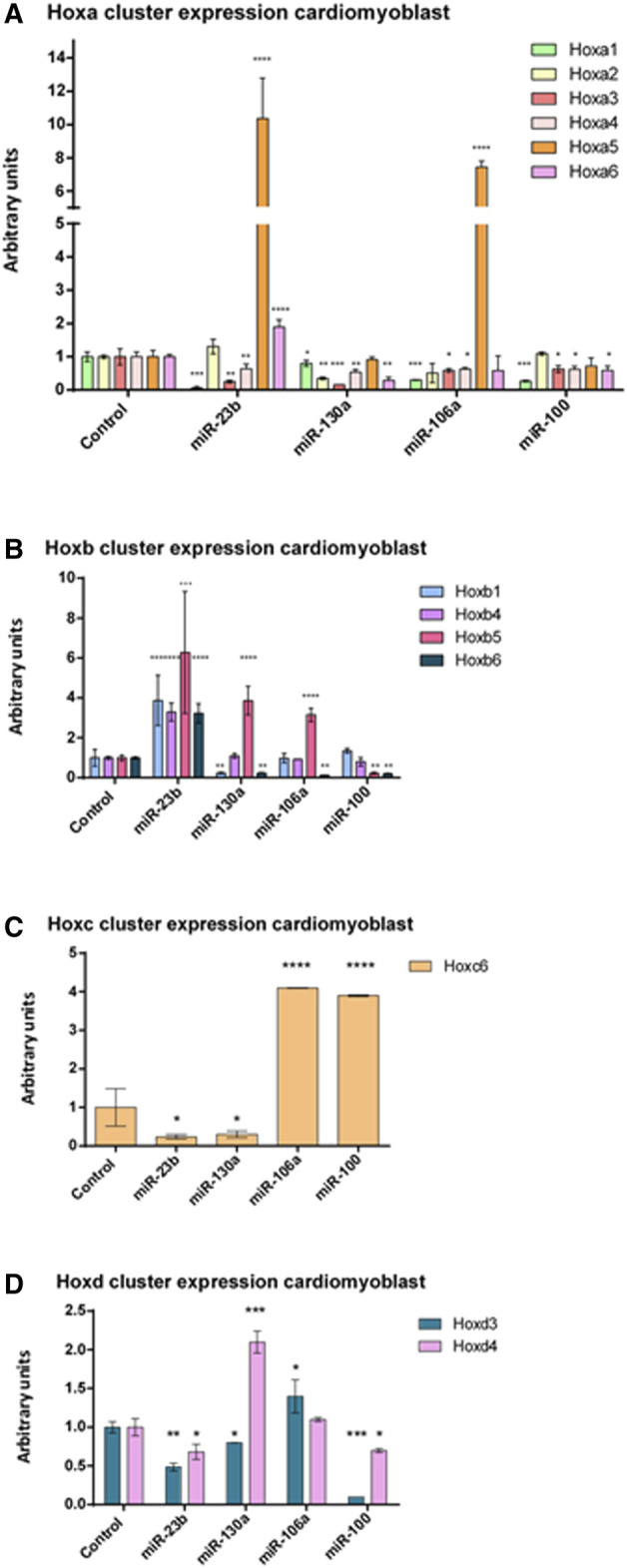
qPCR analysis of Hoxa **(A)**, Hoxb **(B)**, Hoxc **(C)**, and Hoxd **(D)** clusters expression after miR-23b, miR-130a, miR-106a, and miR-100 gain-of-function assays in H9c2 cardiomyoblasts illustrating significant up- and downregulation of these Hox genes. Student’s *t*-test: *p < 0.05, **p < 0.01, ***p < 0.005, ****p < 0.001.

It is to note that H9c2 cardiomyoblasts can be differentiated into cardiomyocytes by administrating retinoic acid (RA) to the culture medium when cells reach a high degree of confluence (Branco et al., 2015). Our expression analyses of Nkx2.5—an early cardiogenic marker—and cardiac troponin T (cTnT)—a late differentiation marker—corroborated that there are differences between H9c2 cardiomyoblasts and cardiomyocytes (Supplementary Figure S1). To assess the possible role of these microRNAs as Hox gene modulators, we performed microRNA gain-of-function assays in cardiomyoblasts (Supplementary Figure S2). As observed in Figure 5 and summarized in Table 1, after gain-of-function experiments in cardiomyoblasts, Hoxa1, Hoxa3, and Hoxa4 were repressed by the four microRNAs studied. Also, Hoxa2 and Hoxa5 were inhibited by miR-130a, and Hoxa6 was downregulated by miR-130a and miR-100. On the other hand, Hoxa5 was significantly upregulated by miR-23b and miR-106a administration. Hoxa6 was also upregulated by miR-23b (Figure 5A). With respect to the Hoxb cluster (Figure 5B, Table 1), different Hox genes were repressed by their respective microRNAs (Hoxb1/miR-130a; Hoxb5/miR-100 and Hoxb6 by mir-130a, miR-106a and miR-100). On the other hand, Hoxb1, Hoxb4, Hoxb5, and Hoxb6 were significantly upregulated by miR-23b. Hoxb5 was also upregulated by miR-130a and miR-106a. Hoxb2 and Hoxb3 were not detected in H9c2 cardiomyoblasts. Within the Hoxc cluster, only Hoxc6 expression was observed, showing a downregulation by miR-23b and miR-130a and upregulation by miR-106a and miR-100 (Figure 5C, Table 1). Finally, in reference to the Hoxd cluster (Figure 5D, Table 1), Hoxd3 and Hoxd4 were repressed by miR-23b and miR-100, and also, Hoxd3 was downregulated by miR-130a. In contrast, Hoxd3 and Hoxd4 were upregulated by miR-106a and miR-130a, respectively. Hoxd1 expression was not detected in H9c2 cardiomyoblasts.

**TABLE 1:** Hox genes modulated by microRNAs in cardiomyoblasts.

	Hoxa1	Hoxa2	Hoxa3	Hoxa4	Hoxa5	Hoxa6	Hoxb1	Hoxb4	Hoxb5	Hoxb6	Hoxc6	Hoxd3	Hoxd4
miR-23b													
miR-130a													
miR-106a													
miR-100													

Diagram illustrating Hox genes modulation exerted by miR-23b, miR-130a, miR-106a and miR-100 after gain-of-function assays in cardiomyoblast cell line (obtained from Figure 5). Green arrows: upregulated. Red arrows: downregulated. Black lines: Do not regulate.

**FIGURE 6:**
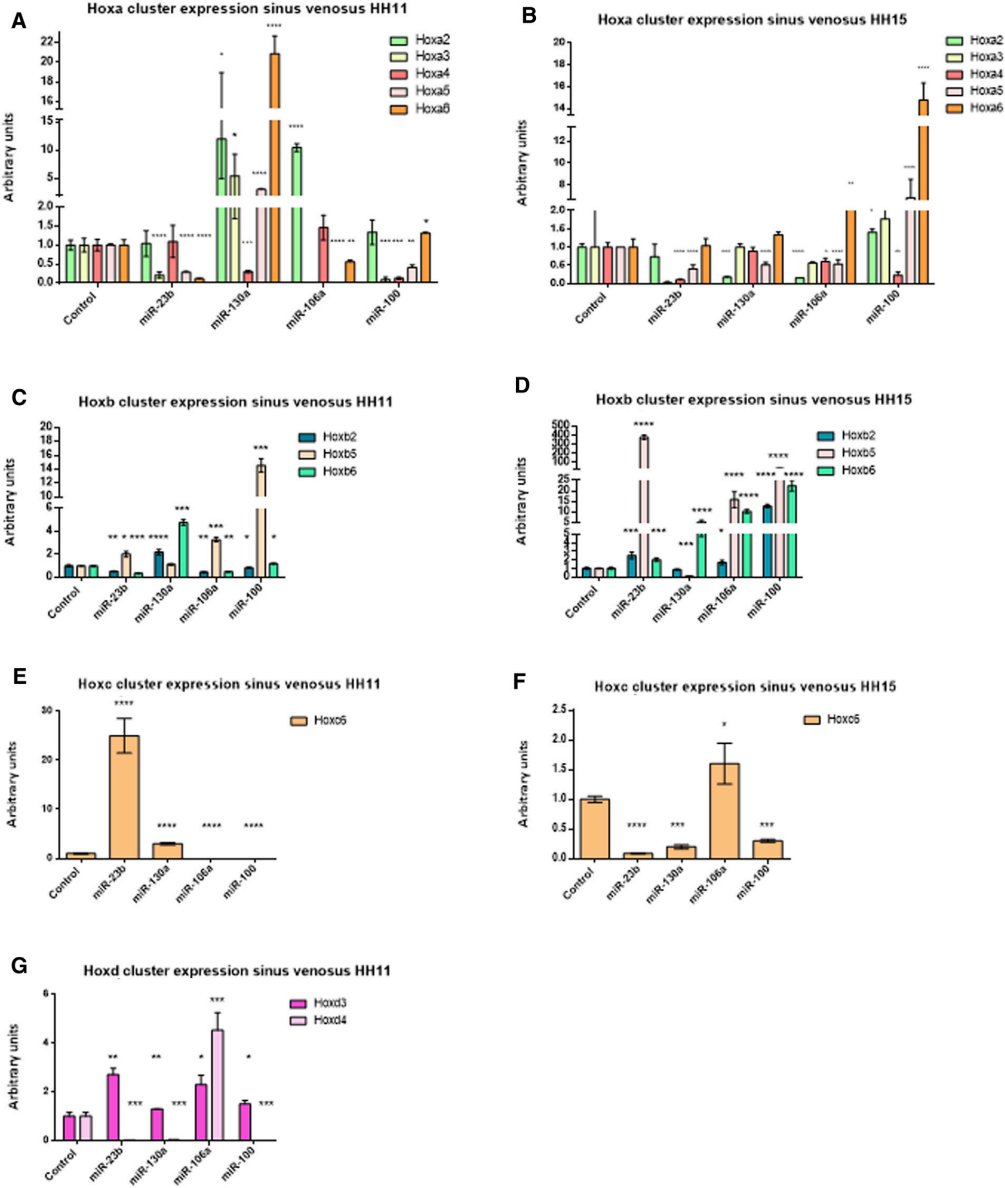
qPCR analysis of the Hoxa cluster at stage HH11 **(A)** and HH15 **(B)**; Hoxb cluster at stage HH11 **(C)** and HH15 **(D)**; Hoxc cluster at stage HH11 **(E)** and HH15 **(F)**, and Hoxd cluster at stage HH11 **(G)** gene expression in *sinus venosus* explants after miR-23b, miR-130a, miR-106a, and miR-100 gain-of-function assays illustrating significant up- and downregulation of these Hox genes. Student’s *t*-test: *p < 0.05, **p < 0.01, ***p < 0.005, ****p < 0.001.

Subsequently, in order to determine the modulation of Hox genes by these microRNAs in the developing venous pole of the heart, we performed gain-of-function experiments in *sinus venosus* explants, at stages HH11 and HH15 (Supplementary Figure S3). Figure 6 and Tables 2, 3 summarize our data, showing that Hoxa3 was downregulated by miR-23b, miR-106a, and miR-100 at stage HH11. Hoxa4 was downregulated by miR-130a and miR-100 at stage HH11 and by miR-23b, miR-106, and miR-100 at HH15 (Figures 6A, B). These data are in line with the results obtained in our gain-of-function experiments in cardiomyoblasts. Additionally, Hoxa5 was repressed by miR-23b, miR-106a, and miR-100 at stage HH11, while it was downregulated by miR-23b, miR-130a, and miR-106a at HH15. Also, Hoxa2 was repressed by miR-130a and miR-106a at HH15, suggesting their involvement in further stages of *sinus venosus* formation. On the other hand, the downregulation of Hoxb6 by miR-106a both in *sinus venosus* explants at HH11 and cardiomyoblasts would suggest a participation in early differentiation. As observed in Figures 6C, D, Hoxb1 and Hoxb4 were not detected in these experiments. Coinciding with the results obtained in cardiomyoblasts, Hoxc6 was downregulated by miR-23b and miR-130a at stage HH15 (Figure 6F) and Hoxd4 was repressed by miR-23b and miR-100 at HH11 (Figure 6G).

**TABLE 2:** Hox genes modulated by microRNAs in the *sinus venosus* at stage HH11.

	Hoxa2	Hoxa3	Hoxa4	Hoxa5	Hoxa6	Hoxb2	Hoxb5	Hoxb6	Hoxc6	Hoxd3	Hoxd4
miR-23b											
miR-130a											
miR-106a											
miR-100											

Diagram illustrating Hox genes modulation exerted by miR-23b, miR-130a, miR-106a and miR-100 after gain-of-function assays in *sinus venosus* explants at stage HH11 (obtained from Figures 6A, C,E, G). Green arrows: upregulated. Red arrows: downregulated. Black lines: Do not regulate.

**TABLE 3:** Hox genes modulated by microRNAs in the *sinus venosus* at stage HH15.

	Hoxa2	Hoxa3	Hoxa4	Hoxa5	Hoxa6	Hoxb2	Hoxb5	Hoxb6	Hoxc6	Hoxd3	Hoxd4
miR-23b										ND	ND
miR-130a										ND	ND
miR-106a										ND	ND
miR-100										ND	ND

Diagram illustrating Hox genes modulation exerted by miR-23b, miR-130a, miR-106a and miR-100 after gain-of-function assays in *sinus venosus* explants at stage HH15 (obtained from Figures 6B, D, F). Green arrows: upregulated. Red arrows: downregulated. Black lines: Do not regulate. ND: no detectable.

**FIGURE 7:**
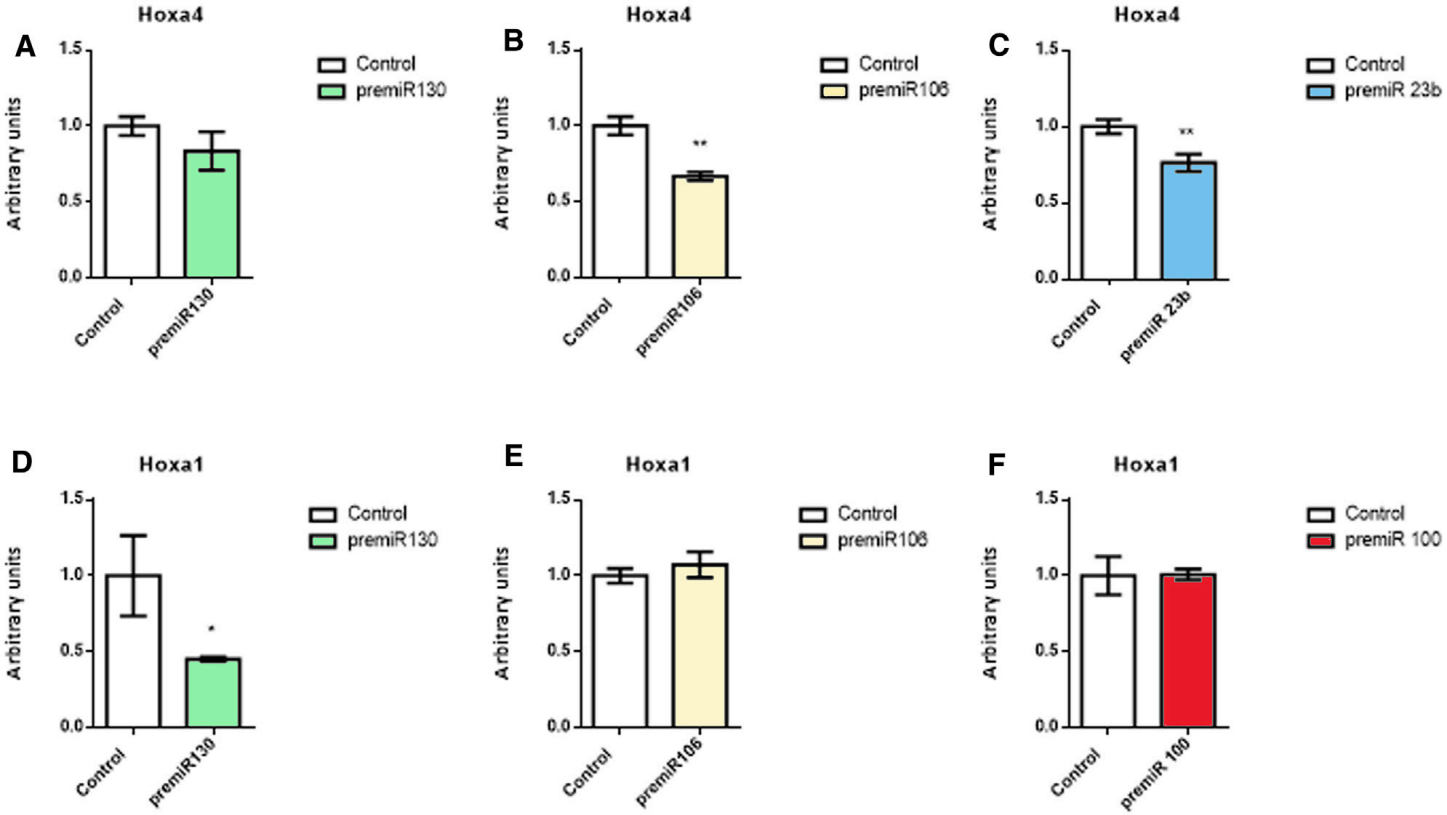
Dual luciferase assays. Representative data of Hoxa4 **(A–C)** and Hoxa1 **(D–F)** 3′UTR luciferase assays after miR-130a, miR-106a, miR-23b, and miR-100 overexpression in 3T3 fibroblasts. Student’s *t*-test: *p < 0.05, **p < 0.01, ***p < 0.005, ****p < 0.001.

Since Hoxa1 and Hoxa4 expressions were modulated tnegatively by those microRNAs analyzed *in vitro* and given that their 3′UTR harbor multiple seed sequences for these microRNAs, we performed dual luciferase biochemical assays to determine whether miR-23b, miR-130a, miR-106a, and miR-100 could directly target either Hoxa4 or Hoxa1 3′UTRs (Figure 7). Our data demonstrated that miR-130a could not interact directly with Hoxa4 3′UTR (Figure 7A), which is in agreement with the fact that this microRNA is not predicted either by mirWalk or TargetScan. Interestingly, transfection with miR-106a and miR-23b significantly decreased Hox4 luciferase levels with respect to control samples (Figures 7B, C), in line with mirWalk predictions (Supplementary Table S1), thus proving a direct interaction between those two microRNAs and Hoxa4 3′UTR. As for Hoxa1 3′UTR (Figures 7D–F), only miR-130a reduced luciferase levels with respect to the control, thus suggesting an indirect repression exerted by miR-23b, miR-106a, and miR-100 on this gene.

## Discussion

Previous studies have demonstrated that a single microRNA may be a crucial regulator both in cardiac development and function. Imbalanced microRNA expression in the progenitor cells of the developing heart might cause congenital and/or structural defects, including altered cell migration and proliferation, as well as inappropriate cell type specification (Pang et al., 2019; Kalayinia et al., 2021). In particular, it has been reported that: 1) miR-23b is upregulated in cardiac hypertrophy, and its over-expression in cardiomyocytes *in vitro* is sufficient to promote hypertrophic growth (Thum et al., 2008; Boureima Oumarou et al., 2019); 2) miR-130a is required for adequate proliferation of cardiac progenitors (Kim et al., 2009), supported by gain-of-function murine experiments, leading to cardiomyocyte proliferation defects as ventricular hypoplasia; 3) miR-106a is significantly upregulated in cardiac hypertrophy, as demonstrated by *in vivo* and *in vitro* analyses (Guan et al., 2016); and 4) although miR-100 function is not relevant to hypertrophic gene expression, its role in cardiac regeneration has been widely demonstrated in zebrafish and mice, and it also plays specific roles in adult isoform cardiac gene regulation (Tarantino et al., 2010; Aguirre et al., 2014).

Since these microRNAs have proved significant in cardiac structural and functional features, we will proceed to discuss their relevance during cardiac development. Previously, the expression profile of miR-23b has been identified in the cardiac tissue of fetal mice from E12.5 to E18.5 (Cao et al., 2012). Also, miR-23b expression has been observed in developing chicks, although restricted to the atrium and the dorsal aorta, since stage HH22. On the other hand, a widespread expression of miR-130a, miR-106a, and miR-100 has been described (Darnell et al., 2006), although cardiac expression of these microRNAs has not been previously described. In our study, we carried out a detailed analysis of miR-23b, miR-130a, miR-106a, and miR-100 expression profiles, from early gastrulation stages to the formation of the early cardiac looping stage. Our results reveal miR-23b, miR-130a, and miR-106a expressions in the primitive streak and the first heart field, maintaining their expressions in both primitive endocardial tubes and cardiac tube formation. In addition, miR-100 expression is observed during cardiac looping stages. Subsequently, these four microRNAs show common expression in specific cardiac structures, including inflow and outflow tracts, as well as the dorsal mesocardium and the proepicardium. This is the first time that the cardiac expression profiles of these four microRNAs have been described, suggesting that they could play crucial roles in multiple cardiac development processes from early stages.

In our study, we show that numerous Hox genes are expressed in the *sinus venosus* at stages HH11 and HH15. These data are relevant since, to date, only the expression of a few Hox genes—Hoxa1, Hoxa3, Hoxa4, Hoxb1, and Hoxb4—has been demonstrated in different species (Searcy and Yutzey, 1998; Makki and Capecchi, 2010; Bertrand et al., 2011; Barak et al., 2012; Roux et al., 2015; Lescroart and Zaffran, 2018; Stefanovic et al., 2020). We have subsequently explored the possibility that those microRNAs studied by ISH, the expression of which is particularly observed in the *sinus venosus*, could recognize 3′UTRs regions of the Hox genes and modulate their expression. Based on *in silico* analysis, we observe that one or more of the microRNAs under study recognized all cranially expressed Hox genes, including all paralogues (Hoxa to Hoxd) from 1 to 6, except Hoxa6. The potential modulation of Hox gene expression is confirmed by the results we obtained from *in vitro* assays in cardiomyoblasts and *ex vivo* assays in *sinus venosus* explants.

The role of retinoic acid (RA) in differentiation and morphogenesis of structures derived from the posterior segment of the heart tube has been widely described (Bertrand et al., 2011; De Bono et al., 2018b). Deficient RA synthesis results in cellular hypoplasia and, consequently, in morphogenetic defects in both the atrium and the *sinus venosus* (Niederreither et al., 1999, 2001). Interestingly, the administration of RA during mouse lung development is sufficient to induce Hoxa4 expression (Packer et al., 1998, 2000). Also, this gene has been recognized in many tissues as a potent inhibitor of cell mobility (Bhatlekar et al., 2017; Cheng et al., 2018). In our study, Hoxa4 provides an interesting example of a Hox gene downregulated by the microRNAs analyzed, as illustrated by both *in vitro* and *ex vivo* assays. In this context, it should be noted that the mobility of the cardiac progenitors is a dynamic and continuous determinant process during heart remodeling and conformation. Similarly, adequate cardiac physiological function requires certain cell subpopulation movement, not yet fully defined, from their origin to other cardiac regions. The repression of Hoxa4 that we observed in the *sinus venosus* at stages HH11 and HH15 reflects a much more complex mechanism, which may be responsible for cell mobility regulation of the different cardiac progenitors, likely involved in the developing venous pole of the heart. Although Hoxa4 modulation by means of microRNAs has not yet been described, some authors have demonstrated a negative modulation of Hoxa5 by miR-130a, after induction of Hoxa5 by RA (Yang et al., 2013). Supporting these data, our luciferase assays reveal that Hoxa4 is repressed by miR-23b and miR-106a, as the consequence of a direct physical interaction exerted between these microRNAs and its 3′UTR region.

On the other hand, we observe that Hoxd3 is upregulated both in the cardiomyoblasts and the *sinus venous* at stage HH11. Previous reports have pointed out that Hoxd3 is not expressed at later stages of cardiogenesis and also that treatment with RA is sufficient to repress its expression (Searcy and Yutzey, 1998), showing an opposite behavior to that of Hoxa4. In agreement with the above, in our study we observed that the expression of Hoxd3 is not detected in the *sinus venosus* at stage HH15. This fact could be a consequence of the specific restricted pattern of the microRNAs—miR23b, miR-130a, miR-106a, and miR-100- during this stage.

In summary, this study shows several novel findings in the field of cardiac development. Our data show a dynamic expression of miR-23b, miR-130a, miR-106a, and miR-100 from early stages of cardiogenesis. We also identify the expression of several Hox genes in the *sinus venosus* at stages HH11 and HH15. Noticeably, we observe that there is a negative modulation of several Hox genes by microRNAs, both in cardiomyoblasts and *sinus venosus*. Finally, the convergent expression of these microRNAs regulating Hox gene expressions in the *sinus venosus*/inflow tract supports the hypothesis of a potential role in differentiation and compartmentalization of the cardiac venous pole.

## Materials and Methods

### Whole-Mount LNA *In Situ* Hybridization (ISH) and Sectioning

Fertilized eggs (Granja Santa Isabel, Córdoba, Spain) were incubated at 38°C in forced-draft, humidified incubators. Embryos were collected at stages HH3 to HH17 (Hamburger and Hamilton, 1951, 1992; Lopez-Sanchez et al., 2005) and fixed overnight at 4°C in 4% PFA, dehydrated in methanol, and stored at −20°C. Embryos were processed for LNA-ISH following our previous procedure (Lopez-Sanchez et al., 2015a) using miR-23b, miR-130a, miR-106a, and miR-100 LNA-labeled microRNA probes (miRCURY LNA™ Detection probe 5′-DIG and 3′-DIG labeled, Exiqon), respectively.

For histology, embryos were dehydrated with an ethanol series, cleared in isopropanol, and processed for paraplast embedding, obtaining 15-μm transverse serial sections.

### H9c2 Cell Culture and microRNAs Transfections

The H9c2 cell line (kindly provided by Dr. Paulo J. Oliveira, Coimbra, Portugal) was cultured in DMEM medium supplemented with 10% foetal bovine serum, 100 U/mL penicillin, and 100 μg/ml streptomycin in 100-cm^2^ culture disks at 37°C in a humidified atmosphere of 5% CO_2_. Cells were fed every 2–3 days. Two sub-cultured condition transfections were performed. Myoblast H9c2 was sub-cultured at 50–60% confluence while induced-cardiomyocyte H9c2 reached 80–90% confluence. H9c2 cells (6 × 10^5^ cells per well) were transfected with microRNA mimics for miR-23b, miR-130a, miR-106a, and miR-100 precursors (Thermo Fisher) as previously described (Branco et al., 2015).

### *Sinus Venosus* Resection and microRNAs Transfections

Groups of embryos were collected at stages HH11 and HH15 and maintained in EBSS (Gibco) at low temperature until manipulation. The *sinus venosus* was resected from the embryos and transfected in hanging drops (Bonet et al., 2015; Dueñas et al., 2020) with microRNA mimics, including miR-23b, miR-130a, miR-106a, and miR-100 precursors, for 24 h at 37°C.

Pre-miRNAs transfection was carried out with Lipofectamine 2000 (Invitrogen) following the manufacturer’s instructions. Negative control explants were treated only with Lipofectamine and were run in parallel.

### RNA Isolation and qRT-PCR

Samples obtained from H9c2 cells and *sinus venosus* explants after microRNA transfections and control samples were subjected to qRT-PCR analysis following MIQE guidelines (Bustin et al., 2009; Bonet et al., 2015; Lozano-Velasco et al., 2015). RNA was extracted and purified by using a ReliaPrep RNA Cell Miniprep System Kit (Promega) according to the manufacturer’s instructions. For mRNA expression measurements, 1 μg of total RNA was used for retro-transcription with a Maxima First Strand cDNA Synthesis Kit for qRT-PCR (Thermo Scientific). Real-time PCR experiments were performed with 2 μL of cDNA, Go Taq qPCR Master Mix (Promega), and corresponding primer sets (Supplementary Table S2). For microRNA expression analyses, 20 ng of total RNA was used for retro-transcription with a universal cDNA Synthesis Kit II (Exiqon), and the resulting cDNA was diluted 1/80. Real-time PCR experiments were performed with 1 μL of diluted cDNA and Go Taq qPCR Master Mix (Promega) as well. All qPCRs were performed using a CFX384TM thermocycler (Bio-Rad) following the manufacturer’s recommendations. The relative expression of each gene was calculated by using Gusb and Gadph as internal controls for mRNA expression analyses and *5S* and *6U* for microRNA expression analyses, respectively (Livak and Schmittgen, 2001). Each PCR reaction was carried out in triplicate and repeated in at least three distinct biological samples to obtain representative means. qPCR data were analyzed using ΔDeltaCt (Deepak et al., 2007).

### Amplification of Hox Genes From *Sinus Venosus* cDNA at Stages HH11–HH15

CDNA from the *sinus venosus* at stages HH11 and HH15 was obtained and processed as described above. For mRNA expression detection, Dream Taq polymerase (2x) and specific primers (Supplementary Table S2) were used, taking Gapdh as the internal loading control.

### Luciferase Assays of 3′UTRs Hox Genes

Hoxa1 and Hoxa4 3′UTR constructs were PCR-amplified from chicken genomic DNA using primers bearing SpeI/HindIII restriction sites and cloned into the pGLuc-Basic vector (New England BioLabs). 3T3 fibroblasts (ATCC) were co-transfected with 100 ng of the Hoxa1 and Hoxa4 luciferase vector, 300 ng of pcLux vector control for internal normalization, and 20 nM of each microRNA. Luciferase activity was measured at 24 h after transfection (Pierce™ Gaussia Luciferase Flash Assay Kit) and normalized to the pcLux vector control (Pierce™ Cypridina Luciferase Flash Assay Kit). Luciferase activity was compared to non-transfected controls. Each luciferase assay was carried out in triplicate and repeated in at least three distinct biological samples to obtain representative assays.

### Statistical Analysis

Student’s *t*-test was used. Significance levels or *p*-values are stated in each corresponding figure, *p* < 0.05 being considered as statistically significant.

## Data Availability

The original contributions presented in the study are included in the article/Supplementary Materials, further inquiries can be directed to the corresponding author.
