# Supplementary material for: Dynamic MicroRNA Expression Profiles During Embryonic Development Provide Novel Insights Into Cardiac Sinus Venosus/Inflow Tract Differentiation
Source: Front Cell Dev Biol. 2022 Jan 11;9:767954. doi: 10.3389/fcell.2021.767954 (PMC8787322; doi:10.3389/fcell.2021.767954)
Supplement: Supplementary file 1 [file Table1.pdf]

Supplementary Table 1

Hox gene	microRNAs predicted by miRWalk	microRNAS predicted by TargetScan
Hoxa1	miR-130a, miR-106a, miR-100	miR-23b, miR-130a, miR-100
Hoxa2	miR-23b	miR-23b, miR-130a, miR-106a
Hoxa3	miR 106a	miR-23b, miR-130a, miR-106a
Hoxa4	miR-23b, miR106a	-
Hoxa5	miR-23b	miR-130a
Hoxa6	-	-
Hoxb1	-	miR-130a
Hoxb2	-	miR-130a
Hoxb3	miR-100	miR-130a
Hoxb4	miR-23b	miR-23b, miR-130a, miR-106a
Hoxb5	miR-100	miR-23b, miR-130a
Hoxb6	-	miR-23b, miR-130a
Hoxc4	-	miR-23b, miR-130a
Hoxc5	miR-23b	-
Hoxc6	miR-23b	.
Hoxd1	-	miR-130a
Hoxd3	miR-130a, miR-106a	miR-106a
Hoxd4	miR-23b	miR-106a

Analysis of Hox genes targeted by each microRNA –miR-23b, miR-130a, miR-106a and miR-100- by using miRWalk and TargetScan predictions.
