# Supplementary material for: Dynamic MicroRNA Expression Profiles During Embryonic Development Provide Novel Insights Into Cardiac Sinus Venosus/Inflow Tract Differentiation
Source: Front Cell Dev Biol. 2022 Jan 11;9:767954. doi: 10.3389/fcell.2021.767954 (PMC8787322; doi:10.3389/fcell.2021.767954)
Supplement: Supplementary file 2 [file Table2.pdf]

Supplementary Table 2A

Gene	Specie		Sequence
Hoxa1	Gallus gallus		ACAACCATGCCCTGTCTCC
			GCTTGGTGGTGAAGTTGGTC
Hoxa2	Gallus gallus		GAAAGAGAAAAAGGCGTCCA
			AAGAGCTGCGTGTTGGTGTA
Hoxa3	Gallus gallus		TTCCCGTGGATGAAAGAGTC
			GTTCTACCAGCTGGGCACTT
Hoxa4	Gallus gallus		GGAAGAAGGACCACAAGCTG
			TCTCCCTCCTTCCATTTCCT
Hoxa5	Gallus gallus		GATACCTGACCCGCAGAAGA
			ACAATGTGAATGGCCAGGAG
Hoxa6	Gallus gallus		TACACGAGCCCTGTTTACCC
			AGAGCGTTCGCAATCTCAAT
Hoxb1	Gallus gallus		AACACCATTCGCACCAACTT
			GGCGAGACCTTCCTTTTCC
Hoxb2	Gallus gallus		AAAGCTACCCAGGCTCCATC
			CAGGAGCTGTGTGTTGGTGT
Hoxb3	Gallus gallus		AAGCAGATTTTCCCCTGGAT
			ACAGGTAGCGGTTGAAGTGG
Hoxb4	Gallus gallus		TCTTGCAAAGAGCCGGTAGT
			CTCGGAGAGGCACAGAGAAT
Hoxb5	Gallus gallus		CACTCCAATAAGCAGCAGCA
			TTTTCCAACTCCAGGGTCTG
Hoxb6	Gallus gallus		GAGAGCGAGGAGCAAAAGTG
			AGGTAGCGGTTGAAGTGGAA
Hoxc4	Gallus gallus		TATCCACCACCTCCACCACT
			TTTTCCAGCTCCAAAACCTG
Hoxc5	Gallus gallus		AGGGCTCTGCAGAAATCAAA
			GCCGGGTGAGGTATCTGTTA
Hoxc6	Gallus gallus		GCCCTCAATTCAACTGCCTA
			ACAGACATCAATCGCACAGG
Hoxd1	Gallus gallus		AGAGGAGGAGCAGAAACCCCAG
			TTTGCACAGGAAGGGAGGAAGAC
Hoxd3	Gallus gallus		CACCTACACGGACCTCTCCT
			CTTCGGGGGCTACACGTAC
Hoxd4	Gallus gallus		TCGAGATAGCGACACACTTTG
			CAGAGCAGCTACCTAGGCGA
Gapdh	Gallus gallus		TGTCCTCTCTGGCAAAGTCC
			TGCCCATTGATCACAAGTTT
GusB	Gallus gallus		CGTACCAGCCACTACCCCTA
			TTATCCCTGCGGATCAGTTC

Primer sequences and corresponding Hox genes for qRT-PCR (*Gallus gallus*).

Supplementary Table 2B

Gene	Specie		Sequence
Hoxa1	Rattus norvegicus		CACCAAGAAGCCTGTCGTTC
			CTGCGGGCTCTTGTTAGGTA
Hoxa2	Rattus norvegicus		ACCCCTGGATGAAGGAGAAG
			GGGGTCTGCAGAGGTACTTG
Hoxa3	Rattus norvegicus		CCACCGTGGGCAAACAAATC
			GGTAGCGGTTGAAGTGATGA
Hoxa4	Rattus norvegicus		GGTGGTGTACCCCTGGATGA
			GACTTGCTGCCGGGTATAGG
Hoxa5	Rattus norvegicus		ACCCACATCAGCAGCAGAG
			TTCTGGGCCACCTATGTTGT
Hoxa6	Rattus norvegicus		ACCGACCGGAAGTACACAAG
			AGGTAGCGGTTGAAGTGGAA
Hoxb1	Rattus norvegicus		ACCCTGCCCTTCAGAACCTA
			CTTGTGGTGAAGTTGGTGCG
Hoxb2	Rattus norvegicus		AAGAAATCCGCCAAGAAACC
			CAGCAGTTGCGTGTTGGTAT
Hoxb3	Rattus norvegicus		ACTCCACCCTCACCAAACAG
			GCTGCGCGCTTGTGTATG
Hoxb4	Rattus norvegicus		AAGTTGCCCAACACCAAGAT
			GATTACCTCCCAGCGACCA
Hoxb5	Rattus norvegicus		AGGGGCAGACTCCACAGATA
			GGGTCAGGTAGCGATTGAAG
Hoxb6	Rattus norvegicus		GACCTACACCCGCTACCAGA
			ACTGAGCTGAGACGCACTGA
Hoxc4	Rattus norvegicus		GCCCATAGTCTACCCTTGGA
			GGGTCAGGTAGCGGTTGTAA
Hoxc5	Rattus norvegicus		AGAGCAGTGGGGAGATCAAA
			TCTGGTAGCGCGTGTAACTG
Hoxc6	Rattus norvegicus		ACAGACCTCAATCGCTCAGG
			GCCGAGTTAGGTAGCGGTTG
Hoxd1	Rattus norvegicus		CAGCACTTTCGAGTGGATGA
			TCTGTTTCATCCTCCGGTTC
Hoxd3	Rattus norvegicus		GGCCCAATGCTTCTAGCTCA
			GTGGAACTCCTTCTCCAGCT
Hoxd4	Rattus norvegicus		GTCGTGGTCTACCCTTGGAT
			ACTTGCTGTCTGGTGTAGGC
Gapdh	Rattus norvegicus		GGGCAAGGTCATCCCTGAG
			GAGGTCCACCACCCTGTTGC
GusB	Rattus norvegicus		CCGTGGAACAGGGAATGAG
			CTCAGGTGTTGTCATCGTCA
Nkx2.5	Rattus norvegicus		ACCGCCCCTACATTTTATCC
			GACAGGTACCGCTGTTGCTT
cTnt	Rattus norvegicus		GGCGTTGGAAATAGATGGAA
			TTATTCCTGACGTGGTGTGG

Primer sequences and corresponding Hox genes for qRT-PCR (*Rattus norvegicus*).
