# Supplementary material for: Dynamic MicroRNA Expression Profiles During Embryonic Development Provide Novel Insights Into Cardiac Sinus Venosus/Inflow Tract Differentiation
Source: Front Cell Dev Biol. 2022 Jan 11;9:767954. doi: 10.3389/fcell.2021.767954 (PMC8787322; doi:10.3389/fcell.2021.767954)
Supplement: Supplementary file 3 [file Image2.pdf]

# Supplementary Figure 2

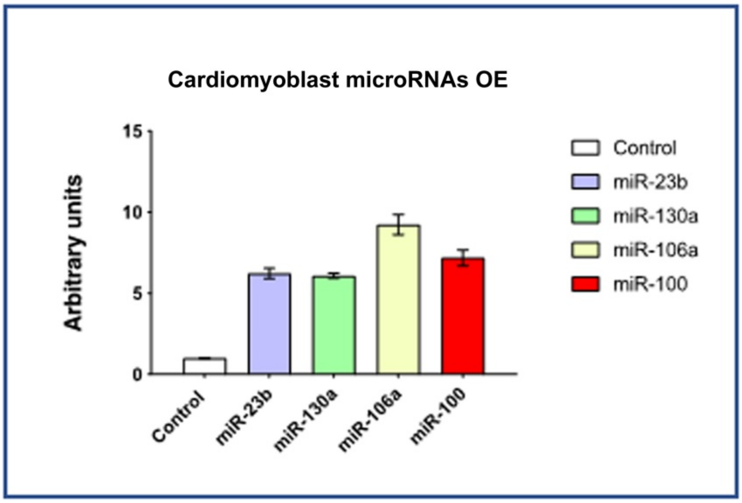


qRT-PCR results validating the over-expression (OE) of analyzed microRNAs in H9c2 cardiomyoblasts transfected cultures.
