# Supplementary material for: Dynamic MicroRNA Expression Profiles During Embryonic Development Provide Novel Insights Into Cardiac Sinus Venosus/Inflow Tract Differentiation
Source: Front Cell Dev Biol. 2022 Jan 11;9:767954. doi: 10.3389/fcell.2021.767954 (PMC8787322; doi:10.3389/fcell.2021.767954)
Supplement: Supplementary file 4 [file Image3.pdf]

Supplementary Figure 3

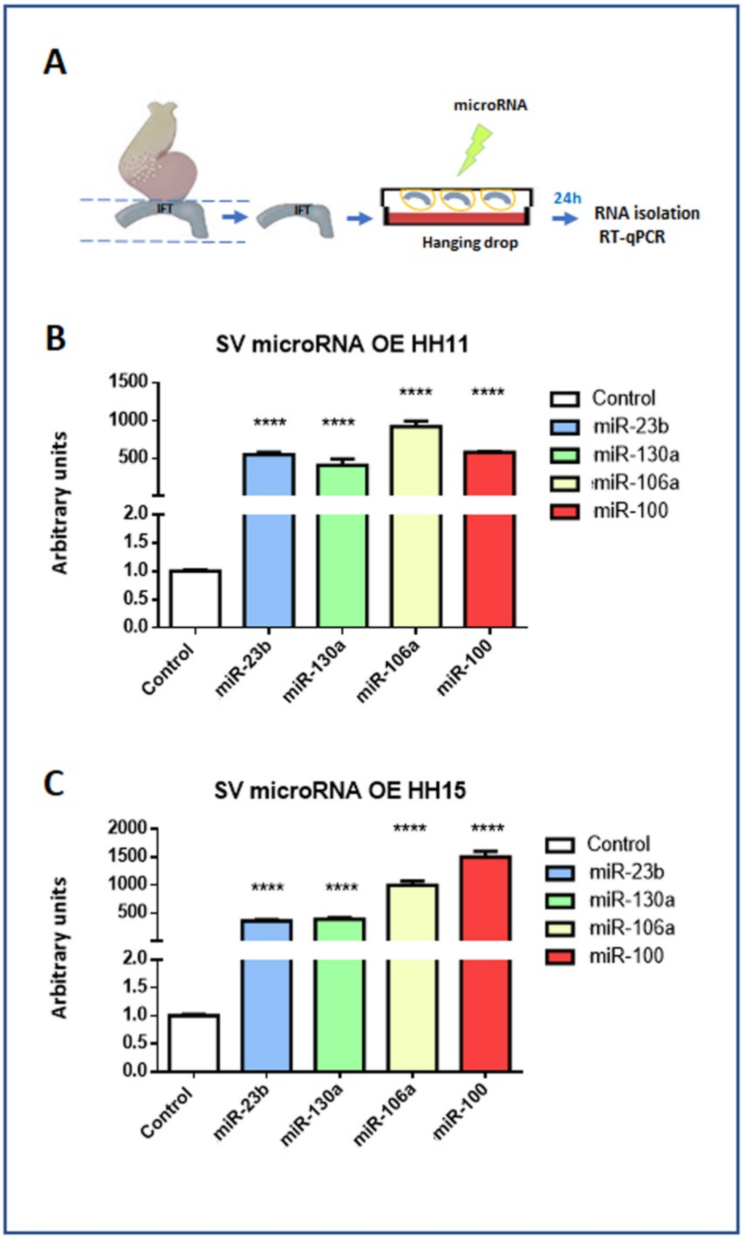

Schematic diagrams (Panel **A**) showing gain-of-function assays in *sinus venosus*/IFT (inflow tract) explants transfected with microRNA in hanging drop during 24h. qRT-PCR results validating the over-expression (OE) of analyzed microRNAs in hanging-drop *sinus venosus* HH11 (Panel **B**) and HH15 (Panel **C**).
