# Supplementary material for: Dynamic MicroRNA Expression Profiles During Embryonic Development Provide Novel Insights Into Cardiac Sinus Venosus/Inflow Tract Differentiation
Source: Front Cell Dev Biol. 2022 Jan 11;9:767954. doi: 10.3389/fcell.2021.767954 (PMC8787322; doi:10.3389/fcell.2021.767954)
Supplement: Supplementary file 5 [file Image1.pdf]

Supplementary Figure 1

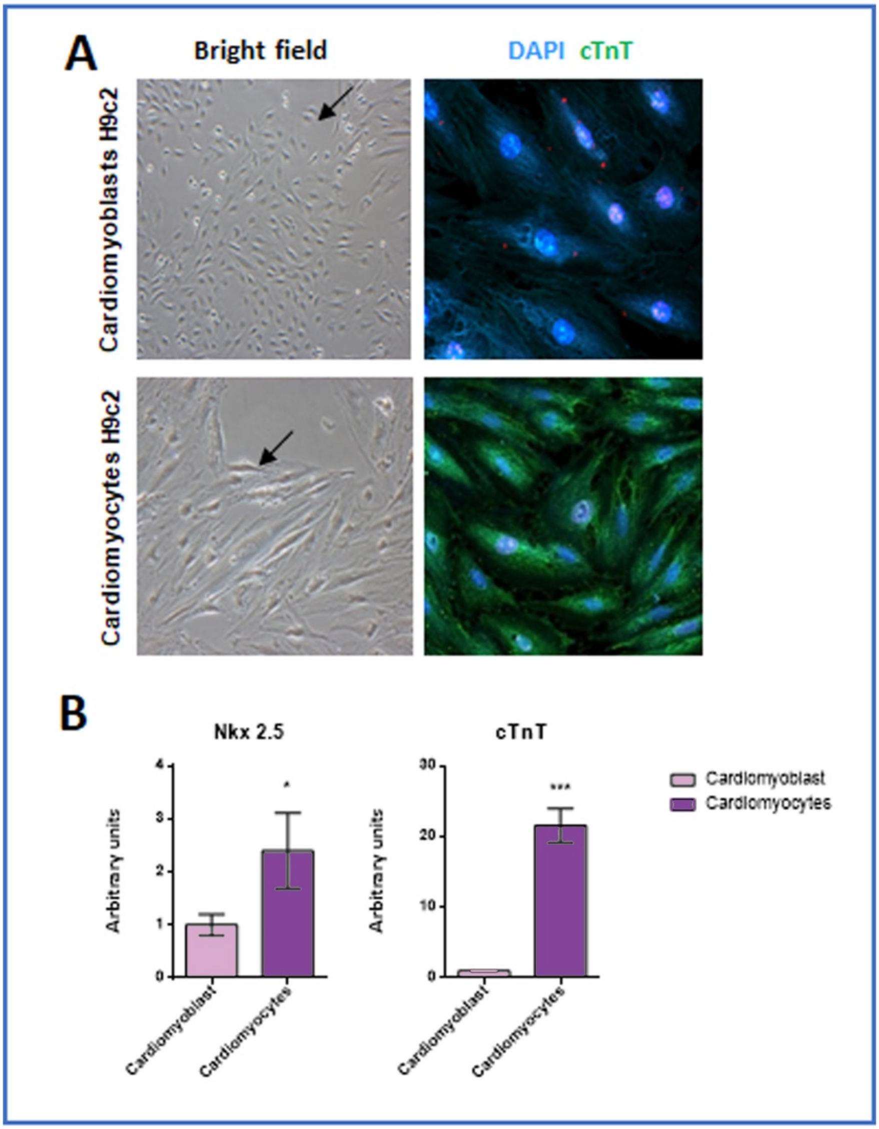

Panel **A** shows morphological differences between cardiomyoblast and cardiomyocyte H9c2 cells (arrows). DAPI has been used as nuclear cell marker (blue colour). Note the expression of cardiac troponin T (cTnT) in the induced-cardiomyocytes (green colour). Panel **B**: qRT-PCR results validating Nkx 2.5 and cTnT markers expression between cardiomyoblasts and cardiomyocytes.
